# Bombyxin II Regulates Glucose Absorption and Glycogen Synthesis through the PI3K Signaling Pathway in HepG2 Cells

**DOI:** 10.1155/2021/6639232

**Published:** 2021-10-18

**Authors:** Hongliang Yang, Hongxia Li, Yang Song, Yujie Sui, Zhenwu Du, Guizhen Zhang

**Affiliations:** ^1^Department of Research Center, Second Hospital of Jilin University, Changchun, China; ^2^Department of Dermatology, The First Hospital of Jilin University, Changchun 130021, China; ^3^Department of Orthopedics, Second Hospital of Jilin University, Changchun, China

## Abstract

Bombyxin, as an insulin-like insect hormone, was discovered in the silkmoth Bombyx mori. It can regulate the metabolism of trehalose and glycogen in Bombyx mori, but whether it has glucose absorption and glycogen synthesis effect on mammalian cells was not clear. BombyxinII (BbxII) and mutant BbxII (mBbxII) genes were cloned into pcDNA3.1(+) vector, respectively; then, gene vectors were transfected into 293FT cells using Lipofectamine 2000. Levels of mRNA and protein expression of BbxII and mBbxII were detected by PCR and Western blot in 293FT cells, respectively. Glucose consumption and glycogenesis were determined by glucose oxidase-peroxidase (GOD-POD) and periodic acid-Schiff (PAS) staining in HepG2 cells; the PI3K signaling pathway was inhibited with wortmannin S1952 in HepG2 cells. Result showed that BbxII and mBbxII genes were being successfully expressed in 293FT cells, respectively. The expression protein of BbxII gene is 10kd pre-bombyxinII, and yet, the expression protein of mBbxII gene is 4kd mature bombyxinII. Only the 4kd bombyxinII showed increased glucose uptake and glycogenesis in HepG2 cells, and the ability of increasing glucose uptake was equal to the human insulin (10 nM). PI3K-wortmannin S1952 inhibitor can decrease the glycogen synthesis induced by bombyxin II protein in HepG2 cells. In conclusion, mature bombyxin II may adjust glucose absorption and glycogen synthesis in HepG2 cells through the PI3K signaling pathway.

## 1. Introduction

Diabetes mellitus (DM) as a chronic metabolic disease has already become one of the major diseases threatening human health in the world. In China, it is reported that the prevalence of diabetes in adults was 10.9%, and the prevalence of prediabetes was 35.7% in 2013 [1]. Development of novel antidiabetic drugs is desired to meet the treatment for increasing diabetes patients.

Bombyxin, the first insulin-like peptide, was discovered in the silkmoth Bombyx mori since 1984 [2]. Bombyxin was produced in four pairs of large neurosecretory cells located in the central back of the moth's brain and released to hemolymph closely associated with feeding [3, 4]. Bombyxin was also called 4K-prothoracicotropic hormone (4K-PTTH), because its MW is about 4400 and can stimulate the prothoracic glands (PGs) to release ecdysone [5]. By analyzing 4K-PTTH purified from Bombyx adult heads revealed an abundance of molecular forms of bombyxin. According to different N-terminal amino acid sequences, bombyxin is divided into three forms, namely, 4K-PTTH-I, II, III; these forms were similar to each other [5].

So far, 4K-PTTH-II as bombyxin-II has been well studied for its structure, genes, distribution, hemolymph titers, secretion control, and physiological functions [6]. The complete amino acid sequence of 4K-PTTH-II such like human insulin consisted of two nonidentical peptide chains (A and B chains); the A and B chains consisted of 20 and 28 amino acid residues and show that they have high similarity with human insulin, which A chain and B chain are 50% and 30%, respectively [5].

Bombyxin-II is an insulin-like insect hormones; its effect on glucose metabolism has become a research focus. In Bombyx mori, several studies revealed bombyxin-II can induce hypotrehalosemia by promoting the hydrolysis of hemolymph trehalose to glucose but not affecting the glucose concentration in the hemolymph by direct bombyxin-II injection [7]. Bombyxin-II can reduce glycogen content of the fat body [7, 8] and promote the proliferation of hematopoietic organ cells and ovarian development [9, 10]. These findings suggest bombyxin-II with hypoglycemic effect and potential to develop into an antidiabetic drug. However, whether bombyxin-II has an effect on glucose metabolism of mammalian cells is not clear.

In this study, we have expressed the 4kd bombyxin-II with eukaryotic expression vector by gene engineering recombination technology in the 293FT cells and observed the effect of bombyxin-II on glucose uptake and glycogen synthesis in HepG2 hepatocellular carcinoma cells in vitro. We confirmed that bombyxin-II can regulate the glucose absorption and glycogen synthesis of human hepatocytes and play the role through the PI3K signaling pathway in vitro.

## 2. Materials and Methods

### 2.1. Bombyxin-II Gene Expression Plasmid Vector

**Figure 1:**
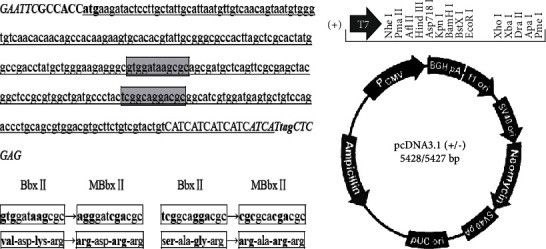
BbxII and MBbxII gene sequence and expression vector. (a) The gene sequence of synthetic BbxII and MBbxII; the italicized capital letter is the enzyme cutting site, the capital plus black is the Kozak sequence, the lowercase underline is the BbxII sequence, the capital underline part is 6 histidine tag sequences, the lowercase plus black box part is the modified gene sequence, and the modified sequence is the MBbxII sequence. (b) The frame map of pcDNA3.1(+) vector. The synthesized BbxII and MBbxII genes were respectively cloned into EcoRI and XhoI digestion sites.

The bombyxin-II (BbxII) gene CDS sequence (NM_001110301) with six histidine tag gene sequences was cloned into pcDNA3.1 plasmid. The vector was named pcDNA3.1-BbxII. This vector can express pre-bombyxin including A and B and C chains in 293FT cells (for more details, see Figure 1).

The mutant bombyxin-II gene sequence with six histidine tag gene sequences was cloned into pcDNA3.1 plasmid. The vector was named pcDNA3.1-MBbxII. This vector can express mature bombyxin including A and B chains in 293FT cells (for more details, see Figure 1).

### 2.2. Reagents

MTT and DMSO were purchased from Tiangen Biochemical Technology Co., Ltd. (China). Glucose assay kit and glycogen staining kit and human insulin were purchased from Nanjing Jiancheng Co., Ltd. (China). High-glucose DMEM medium and phenol red-free 1640 medium and TRIzol and Lipofectamine 2000 were purchased from Thermo (USA). The PI3K inhibitor wortmannin S1952 was purchased from Biyuntian Biotechnology Company (China). Fetal bovine serum was purchased from Tianjin TBD Company (China). Trypsin was purchased from Promega (USA). cDNA first-strand synthesis kit and PCR mix and SYBR PCR mix were purchased from Takara (China). Protein extraction kit and BCA protein detection kit were purchased from Biyuntian Biotechnology Company (China).

### 2.3. Cell Culture

Human 293FT and HepG2 cells were purchased from the Shanghai Institute of Cell Research, Chinese Academy of Sciences (China). 293FT cells were grown in DMEM high-glucose medium (containing 10% fetal bovine serum, 1% nonessential amino acids, 100 g/ml streptomycin, and 100 U/ml penicillin), and HepG2 cells were grown in DMEM high-glucose medium (containing 10% fetal bovine serum, 100 g/ml streptomycin, and 100 U/ml penicillin). Both cells were placed in 37°C, 5% CO_2_ incubator. Medium was changed once at 2-3 days, and cells were digested and passaged when cell fusion was over 80%.

### 2.4. Gene Transfection of 293FT Cells

293FT cells were seeded in a 100 mm diameter cell culture dish and used for gene transfection when the cells were cultured to 90%-95% confluency. The medium without antibiotics was changed at 24 hours before transfection. The cells were divided into four groups: nontransfected gene group, control plasmid group (pcDNA3.1(+)), pcDNA3.1-BbxII/His plasmid group, and pcDNA3.1-MBbxII/His plasmid group. These plasmid vectors were transfected into 293FT cells using Lipofectamine 2000 according to the manual protocol. The procedure is as follows: (1) preparation of solution A that 4.0 *μ*g plasmid was added into 250 *μ*l high-glucose serum-free DMEM medium and mixed gently, and incubated for 5 min at room temperature and (2) preparation of solution B that 10 *μ*l Lipofectamine 2000 was added into 250 *μ*l high-glucose serum-free DMEM medium and mixed gently, and incubated for 5 min at room temperature. Then, add solution B dropwise to the solution A while gently vortexing the solution A-containing tube and incubate the mixture for 10-25 minutes at room temperature to allow the liposome-plasmid complex to form. To discard the old culture medium and replace the new serum-free DMEM medium, then add the above liposome-plasmid transfection mixture dropwise into this cell culture dish. After mixing, the cells were cultured at 37°C, 5% CO_2_ for 6 hours and then replaced by fresh medium with 10% serum.

### 2.5. BbxII Gene mRNA Expression Detection

After gene vectors transfected for 72 hours, the cells were collected, and the total cellular RNA was extracted using TRIzol. Total RNA was reverse transcribed into cDNA using cDNA first-strand synthesis kit, and BbxII mRNA expression was detected by common PCR and real-time quantitative PCR, respectively.

The common PCR procedure is as follows: BbxII detection primer is forward: 5′-GGAATTCGCCACCATGAAGATACTCCTTGCTA-3′, reverse: 5′-GCTCGAGCTAATGATGATGATGATGATGACA-3′. PCR product is 308 bp. Reference gene *β*-actin detection primer is forward: 5′-GGACCTGACTGACTACCTC-3′, reverse: 5′-TCATACTCCTGCTTGCTG-3′. The PCR product was 540 bp. The reaction volume was 20 *μ*l. The reaction conditions were predenatured at 94°C for 5 min, denatured at 94°C for 30 sec, annealed at 55°C for 30 sec, extended at 72°C for 30 sec, for a total of 35 cycles, and finally at 72°C for 10 min. Agarose gel electrophoresis was used to observe PCR products.

The real-time quantitative PCR procedure is as follows: the BbxII detection primer is forward: 5′-TTCGCCACCATGAAGATACTCC-3′, reverse: 5′-TCGGCCATAGTGCGAGCTAAGT-3′. The PCR product is 128 bp. The reference gene GAPDH detection primer is forward: 5′-CCACATCGCTCAGACACCAT-3′, reverse: 5′-ACGGTGCCATGGAATTTGCC-3′. The PCR product was 196 bp and was detected using the SYBR kit. The PCR reaction system was 20 *μ*l. The reaction conditions were predenatured at 94°C for 5 min, denatured at 94°C for 30 sec, annealed at 55°C for 30 sec, and elongated at 68°C for 30 sec, for a total of 40 cycles. PCR reaction was completed in ABI 7500 real-time PCR system (Applied Biosystems) instrument, and the result was analyzed by system software (version 1.40, Applied Biosystems).

### 2.6. Western Blot Analysis of BbxII Protein Expression

After 72 hours of cell transfection, the cells and culture supernatant were harvested, and the protein was extracted using the protein extraction kit and determined by BCA protein detection kit. 50 *μ*g proteins from each sample separation were performed using 15% SDS-PAGE gel and then transferred to a PVDF membrane. After blocking with 5% BSA for 2 h at room temperature, add 1 : 1000 dilution of anti-His-tag mouse monoclonal antibody and anti-GAPDH antibody at 4°C overnight. After washing the membrane, add 1 : 1500 dilution of goat anti-mouse IgG. After 2 hours at room temperature, immunoglobulin detection was performed using ECL luminescent solution. The average optical density analysis of protein bands was performed using ImageJ image analysis software, and the optical density value was calculated using the formula: optical density (OD) value = Log_10_ (255/average gray value). The OD value ratio was determined according to the OD value of the BbxII protein band and the internal reference gene GAPDH protein band for relative quantitative analysis.

### 2.7. MTT Assay for Cell Activity

HepG2 cells were cultured in 96-well plates at 1 × 10^4^/well. Different volumes (100 *μ*l, 50 *μ*l, 25 *μ*l, and 10 *μ*l) of cell culture supernatants of 293FT cells with gene transfection and insulin were added separately in 96-well plates. The 96-well plate was cultured in a 37°C, 5% CO _2_ incubator. After culturing for 72 hours, 20 *μ*l of MTT (3-(4,5-dimethyl-2-thiazolyl)-2,5-diphenyl-2-H-tetrazolium bromide, 5 mg/ml) was added to each well. After 4 hours of incubation, replace the MTT culture medium with 150 *μ*l DMSO. After shaking for 10 min at room temperature, the crystals were sufficiently dissolved, and the absorbance (OD value) of each well was measured at 490 nm by a microplate reader.

### 2.8. Cellular Glucose Consumption Detection

The glucose content was determined by the GOD-POD (glucose oxidase-peroxidase) micromethod according to the instructions. HepG2 cells were cultured in 96-well plates (5 × 10^4^/well), and each group was provided with 3 replicate wells. The 96-well plate was cultured in a 37°C, 5% CO_2_ incubator. After the cells were attached to the wall, the phenol red-free 1640 medium was added, and the supernatant of bombyxin-II gene expression in 293FT cells or insulin was added separately. After 24 hours, the supernatant was collected and 200 *μ*l of phenol red-free 1640 medium was added to each well again. After further culturing for 24 hours, the supernatant was collected. Broth collected supernatants were each extracted 2 *μ*l to test.

Glucose concentration (mmol/L) = (sample tube absorbance value − blank tube absorbance)/(calibration tube absorbance value − blank tube absorbance) × calibration solution (5.55 mmol/L). The glucose content of HepG2 cells was subtracted from the basal glucose content for 24 h and 48 h, and the glucose consumption was calculated at 24 h and 48 h. The 24 h and 48 h glucose consumption is equal to the base glucose content minus the remaining glucose content.

### 2.9. Cell Glycogen Synthesis Detection

Glycogen synthesis of HepG2 cells was detected by PAS staining commercial kit. The PAS staining has been finished as follows: HepG2 cells were cultured in 48-well plates with autoclaved coverslips at a concentration of 4 × 105/well used high-glucose DMEM medium; the cell supernatant of 293FT cells transfected with bombyxin-II gene and insulin was added in each well separately. The well plate was placed in a 37°C, 5% CO_2_ incubator. After cultured for 24 hours, the medium has been replaced with phenol red-free 1640 medium. After cultured for an additional 48 hours, the cell culture medium was discarded and washed once with PBS; the cells were stained successively with solution I, solution II, and solution III involved in PAS staining kit. The staining results were observed by an optical microscope.

The criteria for determining cell staining results according to the degree of staining are as follows: (-) represents that the cytoplasm is colorless, no particles; (+) represents that the cytoplasm showed light red or a small amount of red granules and the number of granules is less than 10; (++) represents that the cytoplasm is red or the number of granules is more than 10; (+++) represents that the cytoplasm is dark red or with coarse red particles and red block; (++++) represents that the cytoplasm is purplish red or has a large red block.

### 2.10. Statistical Analysis

All the data are presented as the mean ± standard error of the mean (SEM). The differences among groups were analyzed by a randomized block design analysis of variance by using SPSS (version 13.0) software. *p* values below 0.05 were considered statistically significant.

## 3. Result

### 3.1. Bombylin II Was Successfully Expressed in 293FT Cells

In the study, we constructed pcDNA3.1-BbxII/His and pcDNA3.1-MBbxII/His vector. The pcDNA3.1-BbxII/His vector may express pre-bombyxin-II in 293FT cells (including A chain, B chain, and C chain). pcDNA3.1-MBbxII/His vector may express mature Bombyx mori (including A chain and B chain) in 293FT cells. The two vectors of the Bombyx-II gene sequence include the restriction enzyme site, the Kozak sequence, the expressed bombyxin-II sequence, 6 histidine tag sequences, and stop codons. The bombyxin-II gene original sequence is expressed by pcDNA3.1-BbxII/His vector. The Bombyx gene sequence expressed in the pcDNA3.1-MBbxII/His vector was transformed, and the A-C and C-B junctions of the Bombyx gene were mutated to the recognition site of the furin protein (Figure 1(a)). Synthesis of the above sequence was successfully recombined into pcDNA3.1(+) plasmid between the EcoRI and xhoI restriction sites (Figure 1(b)).

**Figure 2:**
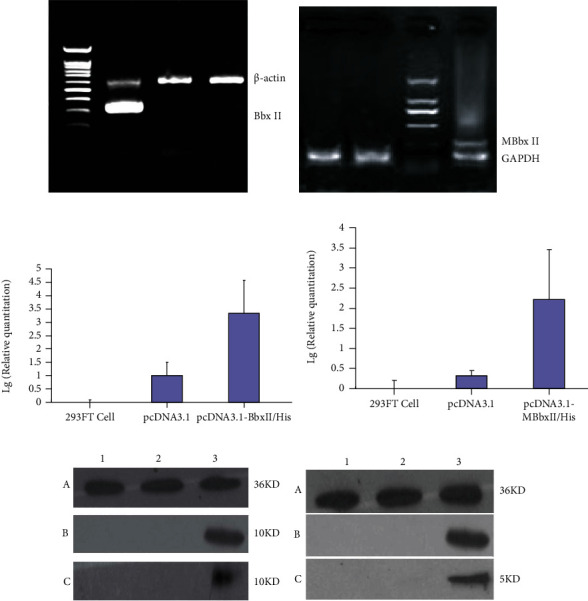
Expression of BbxII and MBbxII gene in 293FT cells. (a) The gel electrophore result of BbxII gene mRNA expression in 293FT cells by RT-PCR. Line 1: DNA marker 1000, line 2: pcDNA3.1-BbxII/His group, line 3: pcDNA3.1(+) group, and line 4: 293FT cells without gene transfection. (b) The gel electrophore result of MBbxII gene mRNA expression in 293FT cells by RT-PCR. Line 1: 293FT cells without gene transfection, line 2: pcDNA3.1(+)group, line 3: DNA marker 2000, and line 4: pcDNA3.1-MBbxII/His group. (c) Relative quantitative analysis of the expression of BbxII mRNA by real-time quantitative PCR. (d) Relative quantitative analysis of the expression of MBbxII mRNA by real-time quantitative PCR. (e) The protein expression of BbxII in 293FT cells and supernatant observed by Western blot. Line 1: 293FT cells without gene transfection group, line 2: pcDNA3.1(+) group, and line 3: pcDNA3.1-BbxII/His group. (A) Intracellular GAPDH protein. (B) Intracellular BbxII protein. (C) BbxII protein in cell culture supernatant. (f) The protein expression of MBbxII in 293FT cells and supernatant observed by Western blot. Line 1: 293FT cells without gene transfection group, line 2: pcDNA3.1(+) group, and line 3: pcDNA3.1-MBbxII/His group. (A) Intracellular GAPDH protein. (B) Intracellular BbxII protein. (C) MBbxII protein in cell culture supernatant.

The gel electrophoresis results of RT-PCR showed that the mRNA expression of BbxII gene in 293FT cells transfecting with pcDNA3.1-BbxII/His plasmid vector and pcDNA3.1-MBbxII/His plasmid vector was significantly higher than that of the untransfected group and control group, respectively (Figures 2(a) and 2(b)). Quantitative analysis of BbxII gene expression by real-time PCR showed that the expression of BbxII gene in the pcDNA3.1-BbxII/His plasmid vector and pcDNA3.1-MBbxII/His plasmid vector groups was significantly higher than that of the untransfected group and control group, respectively (Figures 2(c) and 2(d)).

Western blot protein detection results show that the expression of GAPDH was detected in the untransfected group, pcDNA3.1(+) group, pcDNA3.1-BbxII/His group, and pcDNA3.1-MBbxII/His group, and the molecular weight was 36 kD. There was no BbxII and MBbxII protein expression in the untransfected group and pcDNA3.1 group. The expression of BbxII protein was observed in the pcDNA3.1-BbxII/His plasmid vector transfection group regardless of the cell or supernatant, and the molecular weight was 10 kD (Figure 2(e)). The expression of MBbxII protein was observed in the pcDNA3.1-MBbxII/His plasmid vector transfection group regardless of the cell or supernatant, and the molecular weight was 5 kD (Figure 2(f)).

### 3.2. Effect of BbxII Gene Transfection Supernatant on Proliferation of HepG2 Cells

**Figure 3:**
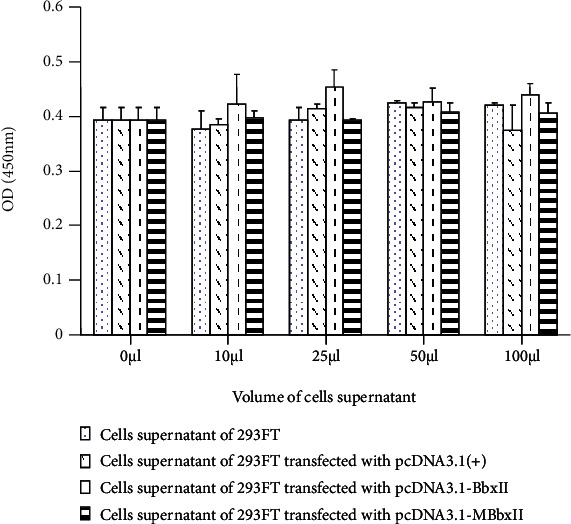
The effect of cell supernatants of 293FT cells transfected with BbxII gene on growth of HepG2 cells.

Compared with the normal gene untransfected group and the control group, the BbxII gene transfection product and MBbxII gene transfection product did not promote cell proliferation in HepG2 (Figure 3).

### 3.3. Effect of Bombyxin II Gene Transfection Supernatant on Glucose Uptake and Glycogenesis in HepG2 Cells

**Figure 4:**
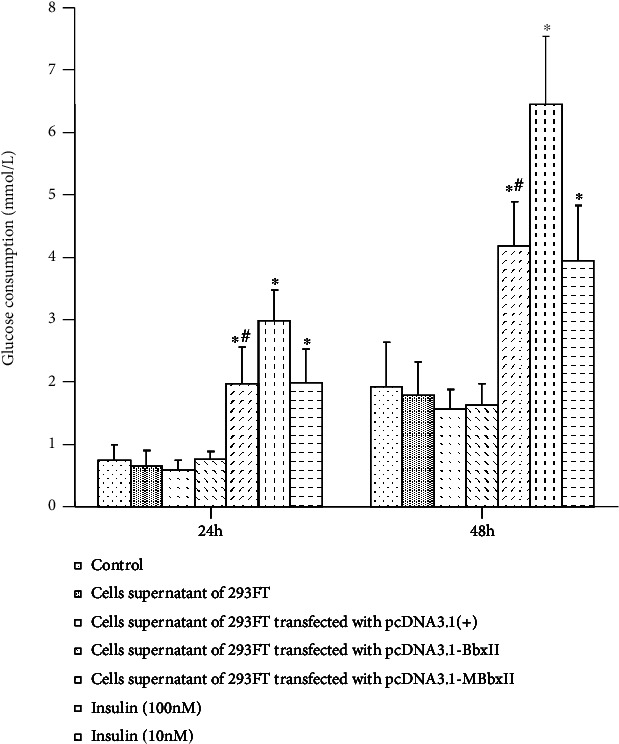
The effect of cell supernatants of 293FT cells transfected with BbxII gene on glucose uptake in HepG2 cells vs. the normal group^∗^, *p* < 0.01; vs. the insulin group^#^ (100 nm), *p* < 0.01.

Compared with the normal group, MBbxII gene transfection of 293FT cell supernatants, and the human insulin action group (100 nM, 10 nM), glucose uptake was increased in HepG2 cells, and the difference was statistically significant (*p* < 0.01). However, the glucose uptake ability of the MBbx II gene-transfected 293FT cell supernatant group was lower than that of the human insulin (100 nM) group; the difference was significant (*p* < 0.01); comparable with the human insulin (10 nM) group, the difference was not significant (*p* > 0.05). Meanwhile, compared with the normal group, there was no significant difference in the glucose uptake capacity of HepG2 cells in the BbxII gene-transfected 293FT cell supernatant group (Figure 4).

Compared with the normal group, the glycogen synthesis in HepG2 cells with the MBbxII gene supernatant group and the human insulin group (10 nM) have significantly increased (*p* < 0.05), respectively. However, the glycogen synthesis of HepG2 cells in the BbxII gene transfection supernatant group did not increase significantly (*p* > 0.05).

**Figure 5:**
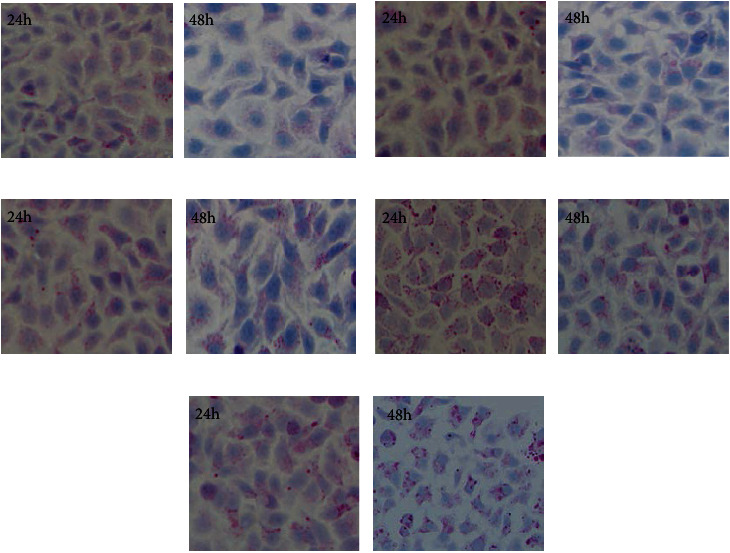
Effects of cell supernatants of 293FT transfected with BbxII/MBbxII gene on glycogen synthesis in HepG2 cells (PAS staining, 400x). (a) Cell supernatants of 293FT. (b) Cell supernatants of 293FT transfected with pcDNA3.1(+)group. (c) Cell supernatant of 293FT transfected with pcDNA3.1-BbxII. (d) Cell supernatant of 293FT transfected with pcDNA3.1-mBbxII. (e) Insulin group (10 nM).

The protein expressed by BbxII gene-transfected 293FT cells did not promote the glycogen synthesis of HepG2 cells. The protein expressed by MBbx II gene-transfected 293FT cells promotes glycogen synthesis in HepG2 cells with an insulin-like action (Figure 5).

### 3.4. Coeffect of MBbxII Gene Transfection Supernatant and PI3K Inhibitor on Glycogen Synthesis of HepG2 Cells

**Figure 6:**
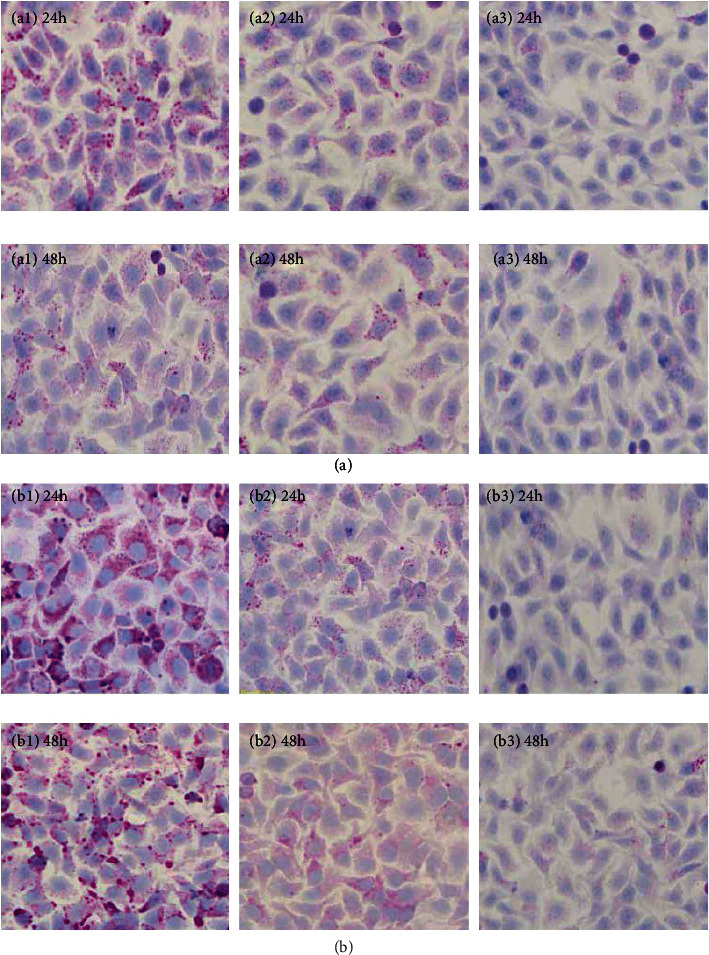
Effects of glycogen synthesis in HepG2 cells with MBbxII transfection supernatant and PI3K inhibitor together (PAS staining) (400x). (a1) Group of MBbxII transfection supernatant. (a2) Group of MBbxII transfection supernatant and wortmannin (5 *μ*M) together. (a3) Group of MBbxII transfection supernatant and wortmannin (10 *μ*M) together. (b1) INS group (10 nM). (b2) Group of INS (10 nM)+wortmannin (5 *μ*M). (b3) Group of INS (10 nM)+wortmannin (10 *μ*M).

Compared with the MBbxII gene transfection supernatant group, the glycogen synthesis of HepG2 cells in the MBbxII+wortmannin (5 *μ*M) treatment group and the MBbxII+wortmannin (10 *μ*M) treatment group decreased; the difference was significant (*p* < 0.05). The results showed that the glycogen synthesis of HepG2 cells was inhibited, and the number of glycogen-positive cells and the staining intensity in HepG2 cells decreased with the increase of I3K inhibitor concentration. This is consistent with the results of glycogen synthesis in HepG2 cells in the INS (10 nM) group, the INS (10 nM)+wortmannin (5 *μ*M) group, and the INS (10 nM)+wortmannin (10 *μ*M) group. The above results indicated that the 293FT cells transfected with the mutant bombyxin-II gene (MBbxII) expressed the mature bombyxin-II protein, which not only promoted glucose uptake by HepG2 cells but also promoted glycogen synthesis (Figure 6).

## 4. Discussion

The present study was aimed at determining whether bombyxin-II has a glucose metabolism effect on mammalian cells. The main finding of our investigation was the 4kd mature bombyxin II not 10kd pre-bombyxin II like the human insulin has the ability of increased glucose uptake and glycogenesis in HepG2 cell. The function of glucose uptake and glycogenesis regulated by mature bombyxin-II may play a role in the PI3K pathway.

Bombyxin II genes encode polypeptides similar to human, which consist of the signal peptide, B-chain, C-peptide, and A-chain; the expression of bombyxin II has the same mechanism as that of insulin: first, the gene is transcribed, then the bombyxin precursor is translated, and the structure is signal peptide (S57)-B chain (b84)-2 basic amino acid residues-C peptide (C63)-2 basic amino acid residues-a chain (A60). Then, the signal peptide is removed to form an in chain disulfide bond and two interchain disulfide bonds called pre-bombyxin. Finally, C peptide is cut; mature pre-bombyxin only including A and B chains was formed [11]. It has not been reported whether both pre-bombyxin and mature bombyxin have effects on the regulation of glucose metabolism in eukaryotic cells. Therefore, in our study, two expression vectors were constructed to express the pre-bombyxin and mature pre-bombyxin. In order to improve the level of gene expression, 293FT human embryonic kidney cells which express SV40 large T antigen were used as host cells. Because 293FT cell is nonendocrine cell and is not a peptidase, the normal bombyxin II gene can only express the pre-bombyxin, not the mature pre-bombyxin. Therefore, we remake the bombyxin II by the junction of pro-bombyxins A-C and C-B into the recognition site of furin protein, so that it can be recognized by furin in nonendocrine cells [12], so as to remove C peptide and form mature bombyxin. Although it has been reported that bombyxin antibody has been used in some research [13], no commercial antibody has been sold. In order to evaluate the expression of silkworm fibroin in cells, we added six histidine tags (a commonly used protein tag) to the downstream of bombyxin protein and indirectly determined the expression of bombyxin by detecting the histidine tag expression (gene sequence and vector; see Figure 1).

In the process of human insulin regulating blood glucose, liver is the most important target organ. The liver can keep the blood glucose stable by controlling the synthesis and decomposition of glycogen and the control of glycogenesis. Glucose uptake and glycogen synthesis are the two most important indexes in the study of glucose metabolism. HepG2 cells, a hepatoma cell line used in this study, are derived from hepatocytes, which retain many characteristics of hepatocytes and have insulin receptors on their surface. They are commonly used as model cells in the study of liver glucose metabolism [14–16]. Therefore, HepG2 cells were used as the target cells to study the effect of silkworm fibroin on human cell glucose metabolism.

In the study, we found that bombyxin II promoted glycogen synthesis in HepG2 cells, while bombyxin II reduced the glycogen content in the fat body and concurrently raised the percentage of active glycogen phosphorylase in insects [7], which suggested that Bombyx mori has different effects on glycogen synthesis in insects and mammalian cells. The main way that human insulin stimulates glycogen synthesis in hepatocytes is through PI3K/Akt enzyme system downstream of insulin receptor [17–19]. At present, the signaling pathway for the action of bombyxin is not completely clear. Some studies referring to the mammalian PI3K pathway showed that bombyxin II and bovine insulin transmitted signal information which did not use the same receptor [20, 21]. The latest research progress on the upstream gene of the silkworm insulin signaling pathway showed that there was a corresponding signaling system in silkworm, which is an evolutionarily conservative signaling pathway, and its key role in life span regulation is also evolutionarily conservative, especially Akt sequence is the most conservative [22, 23].

There are several limitations in this study. First, because commercial bombyxin II detection antibody cannot be obtained in the experiment, we had to express bombyxin II protein which fused with six histamine tags and then to determine indirectly the expression of bombyxin II in 293FT cells by detecting the expression of six histidine tags by Western blot. Second, considering the loss of protein biological activity after protein concentration, we had not concentrated and purified the bombyxin II protein expressed in the culture supernatant of 293FT cell, and the cell supernatants expressing bombyxin II have been acted directly on HepG-2 cells which used to observe the biological activity of bombyxin II; these may make a divergence to verify the real biological activity of bombyxin II. Finally, to investigate the effect of bombyxin II on the PI3K/Akt pathway in HepG-2 cells, only the inhibitor blocking method has been used; there are a little deficiency in the evidence.

## 5. Conclusion

Through this part of experimental research, it is confirmed that the mature bombyxin II protein expressed by eukaryotic cells can promote the glucose uptake and glycogenesis of HepG2 cells and preliminarily explore the regulation of glycogen synthesis by bombyxin II through the PI3K/Akt pathway. The results of this study confirmed for the first time that bombyxin II has the function of glucose uptake and glycogenesis regulation on mammalian cells, which laid the experimental and theoretical foundation for the development of a new antidiabetic drug.

## Data Availability

All data sets used or analysed or generated during the current study are included in this article. The analyzed data are available from the corresponding authors on reasonable request.
